# Spectral Normalized CycleGAN with Application in Semisupervised Semantic Segmentation of Sonar Images

**DOI:** 10.1155/2022/1274260

**Published:** 2022-04-28

**Authors:** Zhisheng Zhang, Jinsong Tang, Heping Zhong, Haoran Wu, Peng Zhang, Mingqiang Ning

**Affiliations:** Institute of Electronic Engineering, Naval University of Engineering, Wuhan, China

## Abstract

The effectiveness of CycleGAN is demonstrated to outperform recent approaches for semisupervised semantic segmentation on public segmentation benchmarks. In contrast to analog images, however, the acoustic images are unbalanced and often exhibit speckle noise. As a consequence, CycleGAN is prone to mode-collapse and cannot retain target details when applied directly to the sonar image dataset. To address this problem, a spectral normalized CycleGAN network is presented, which applies spectral normalization to both generators and discriminators to stabilize the training of GANs. Without using a pretrained model, the experimental results demonstrate that our simple yet effective method helps to achieve reasonably accurate sonar targets segmentation results.

## 1. Introduction

Compared with real aperture side-scan sonar, synthetic aperture sonar (SAS) has higher hydrographic surveying and charting speed and can produce higher resolution images [1–3]. The accurate detection and identification of underwater targets in synthetic aperture sonar continue as a significant issue [4–6]. However, according to the principle of imaging by backprojection [2], images of the same underwater target obtained from different views are different and complex in shape and contour [7], which is hard to be labeled by a supervised detection method [8]. By contrast, semantic segmentation labels mark the outline of the target on the image, which excludes the background area. Thus, the accurate result of semantic segmentation is significant for identifying underwater targets in synthetic aperture sonar and estimating their type, location, scale, direction, and so on [9, 10].

In recent years, deep convolutional neural networks (DCNNs) have been widely used in acoustic image processing. Supervised learning is a generally accepted method for acoustic image semantic segmentation [11]. Combined with specific modules based on the characteristics of sonar images, the improved network can achieve better segmentation results than the original structure. For instance, recurrent residual convolutional neural network (R^2^CNN) is combined with the self-guidance module to help discriminate whether the input image is the segmentation result or the ground truth label [12]. FCN is combined with dilated convolution, dense module, and inception [13]. This method can decrease the parameters of the network and speed up segmentation. Receptive field block and attention search function were integrated into the residual convolutional neural network. This model can help enhance the contrast between the underwater target and the background [14].

However, the main reason for limiting its application to sonar image semantic segmentation is that supervised learning requires a large amount of pixel-level labeled data, which is time-consuming. Besides, a large amount of training data are often unavailable as actual experiments are very expensive and often limited in scale. Thus, semisupervised learning is an important area of research to overcome the problem of limited labeled data. To our knowledge, little work has been done to investigate semisupervised learning for sonar image semantic segmentation.

As soon as the generative adversarial network (GAN) is proposed [15], it is widely used in the field of semisupervised learning. For example, SGAN (semisupervised GAN) [16] is used for multiobjective classification, CC-GAN (context-conditional GAN) [17] can generate oil painting images, and BUS-GAN [18] is applied to improve the segmentation quality of breast lesions from ultrasound images. Recently, the CycleGAN model [19] has become the mainstream choice of image style conversion between domains because it reduces the limitation of image pairing in the training process. It can be applied to semisupervised semantic segmentation by learning a bidirectional mapping from unlabeled real images to available ground truth labels. Jiang et al. firstly exploited this capability to transfer CT to MRI for lung cancer segmentation [20]. Mondal et al. leveraged cycle-consistency loss, which preserves critical attributes between the input and the transformed image to add an unsupervised regularization effect that boosts the segmentation performance when labeled data are limited [21]. His experiments were conducted on three different public semantic segmentation benchmarks: PASCAL VOC 2012 [22], Cityscapes [23], and the Automated Cardiac Diagnosis Challenge (ACDC) [24], whose accuracy is proved better than the traditional adversarial learning method.

However, it is found that CycleGAN tends to generate the same type of segmentation results (mode-collapse) and fails to preserve targets' details for the case of the scarcity and imbalance of sonar image target samples. It is demonstrated by previous research that constraining the Lipschitz constant of the discriminator mapping function *f*(·) can stabilize the training of GANs (L-constraint). The first method to satisfy L-constraint was proposed by WGAN: gradient penalty item was added to the discriminator loss function. The disadvantage of this method is that it can only approximately satisfy the L-constraint only if the number of categories in the training sample is small. The spectral normalization was proposed to limit the Lipschitz constant of the discriminator by limiting the spectral norm of each neural network layer. It can satisfy the L-constraint accurately and does not need the additional hyperparameters tuning. Thus, compared with other normalization techniques, the computation of spectral normalization is relatively tiny. Therefore, it is reasonable to apply spectral normalization to both generators and discriminators.

The main contributions of this paper are as follows:

1. We refine Mondal's [21] semisupervised model and validate its efficiency for two acoustic image datasets. To our knowledge, this is the first investigation applying semisupervised learning for acoustic image segmentation.
2. The spectral normalization is applied to both generator and discriminator to improve the training stability of the CycleGAN.
3. We make two sonar image datasets, SCTDI and SCTDII. SCTDI contains 300 images of three types of targets (shipwreck, aircraft wreckage, and victims). SCTDII contains 800 images of the tiny targets. All images have a fixed resolution of 320 × 320 and 9.6 bits per pixel.

## 2. Related Work

In this section, we review work related to semisupervised segmentation concerning three different aspects-The Application of CycleGAN, Techniques to Stabilize Training of GAN, and Recent Work in Semisupervised Semantic Segmentation.

### 2.1. The Application of CycleGAN

One of the applications of CycleGAN is image synthesis, which is widely used in medical imaging data augmentation.

Image synthesis refers to the mapping between different domains. The image domain in the medical field includes CT images and MRI images. Hiasa et al. [25] proposed a CT to MRI synthesis method using CycleGAN. They extended the CycleGAN approach by adding the gradient consistency loss to improve the accuracy at the boundaries. Huo et al. [26] proposed a novel end-to-end synthesis and segmentation network (EssNet). It can achieve the unpaired MRI to CT image synthesis and CT splenomegaly segmentation simultaneously. Without using manual labels on CT, it can alleviate the manual efforts.

Apart from data augmentation for medical images, CycleGAN can also be employed as a semantic segmentation network and detection framework in remote sensing images.

Dong et al. [27] estimated both segmentation results and monocular depth of three-dimensional (3D) images using CycleGAN, which is meaningful for the study of augmented reality (AR) and autonomous driving applications. Mondal et al. [21] proposed a strategy that enforces cycle consistency to learn a bidirectional mapping between unlabeled real images and real labels. Experiments on three different public segmentation benchmarks (PASCAL VOC 2012, Cityscapes, and ACDC) demonstrate the effectiveness of the proposed method, which outperforms recent approaches based on adversarial learning for semisupervised segmentation.

In the remote sensing images detection field, CycleGAN has been proven generally accepted methods for domain adaptation. For instance, CycleGAN is used to mitigate multisensor differences in a CNN-based unsupervised multiplechange detection approach proposed by Saha et al. [28]. Soto Vega et al. [29] applied the domain adaptation ability of CycleGAN to change detection tasks. This framework can employ previously trained classifiers for new data without a significant drop in classification accuracy. Yang et al. [30] proposed a change detection framework based on selective adversarial adaptation. Adversarial learning further reduces the distribution discrepancy between the target and selected source samples. They prove that not only the positive transfer is enhanced but also the negative transfer is alleviated.

In summary, it has been shown from this review the wide use of CycleGAN's domain adaption ability, which is applied to semisupervised sonar image semantic segmentation task in our work.

### 2.2. Techniques to Stabilize Training of GAN

Goodfellow et al. [15] hold the view that if both the generator and discriminator are powerful enough to approximate any real-valued function. However, GANs can be hard to train, and in practice, it is often observed that gradient descent-based GAN optimization leads to divergence and mode-collapse. A possible explanation for this might be that the network does not satisfy L-constraint.

Researchers have tried to address this instability and improve generators through several techniques. Energy-based GAN [31] and Wasserstein GAN [32] attempt to modify the objective function to improve the quality of gradients. Neyshabur et al. [33] proposed stabilizing GAN training with multiple random projections, namely, training a single generator simultaneously against an array of discriminators, which shows only a low-dimensional projection of the data. Salimans et al. [34] proposed virtual batch-normalization and semisupervised learning to provide additional supervision to the generator.

In this paper, we use spectral normalization [34] to stabilize the training of CycleGAN for semisupervised learning.

### 2.3. Recent Work in Semisupervised Semantic Segmentation

Compared with supervised learning, semisupervised learning can achieve satisfying performance with a small set of labeled data. Quantitative research is generally associated with consistency regularization and has yielded ground breaking results in semisupervised classification problems.

French et al. [35] investigated the conditions that can allow consistency regularization to operate in semisupervised semantic segmentation. Lai et al. [36] presented the context-aware consistency to address the problem that semisupervised models overly rely on the contexts available in the training data. Gurubisic et al. [37] presented a method with one-way consistency for practical real-time applications.

In this paper, we enforce cycle-consistency to achieve satisfying segmentation results.

## 3. Methodology

In this section, domain mapping is firstly introduced to illustrate the unpaired domain adaptation ability of CycleGAN. The loss function is secondly used to describe the optimization goals of the CycleGAN in the semantic segmentation task. Last, it explains why the spectral normalization method applied to both the generator and discriminator of CycleGAN can stabilize the training.

### 3.1. Domain Mapping

**Figure 1:**
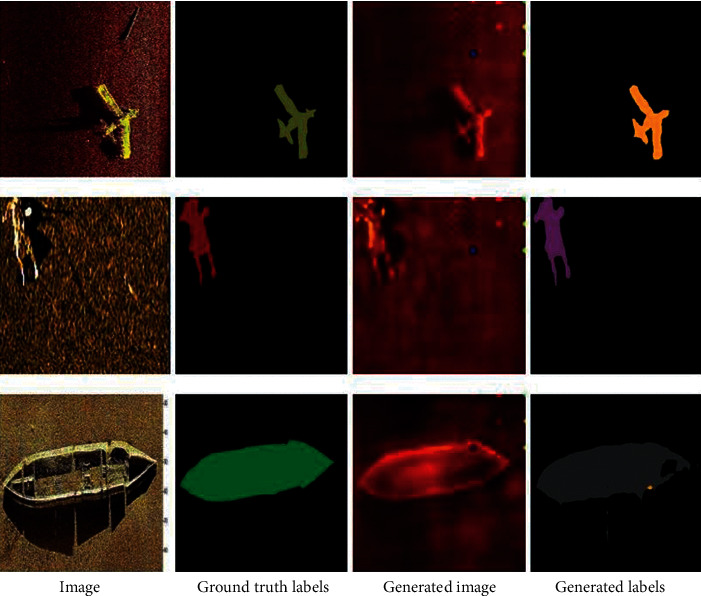
Examples of images, ground truth labels, generated images, and generated labels obtained for three targets: Plane (top row), Person (middle row), and Ship (bottom row).

The domain adaption ability has been explained clearly by [19]. In our work, the source domain refers to sonar images, including real images and generated images. In respect, the target domain refers to labels, including ground truth labels and generated labels.  Figure 1 shows examples of real images (first column), ground truth labels (second column), generated images (third column), and generated labels (fourth column) obtained for the three targets used in our experiments. The different palettes are used to distinguish them for convenience.

**Figure 2:**
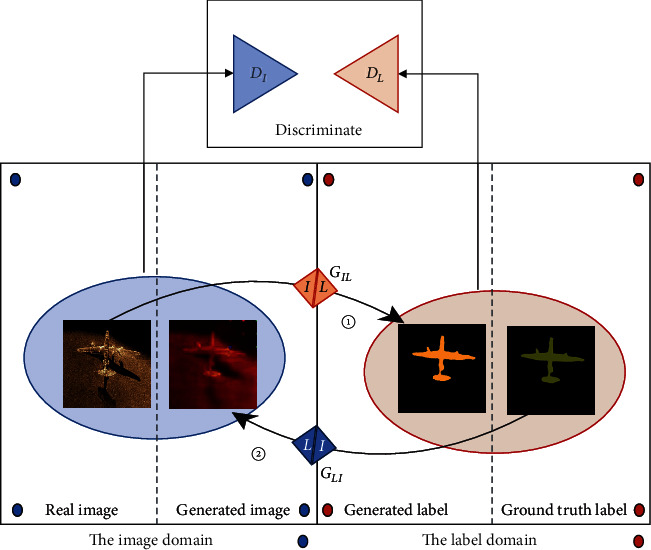
Unidirectional mapping from real image to generated label and from ground truth label to generated image.

**Figure 3:**
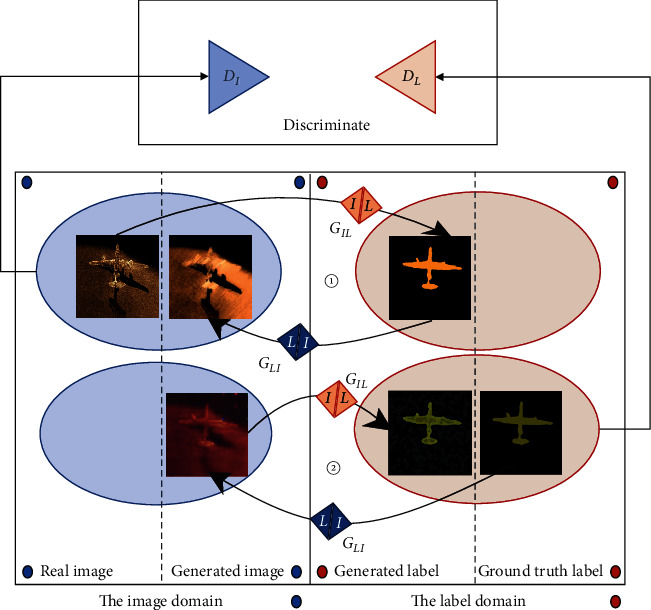
Circular mapping from the real image back to the image domain and ground truth label back to the label domain.

The types of domain mappings can be divided into unidirectional mappings (Figure 2) and circular mappings (Figure 3). The first unidirectional mappings from sonar images to generated labels refer to sonar image semantic segmentation. The circular mappings enforce cycle consistency as the regularization to enhance the semisupervised semantic segmentation performance.

### 3.2. Loss Functions

The data of the semisupervised dataset include three types such as labeled images (*𝒳*_ℒ_), unlabeled images (*𝒳*_*u*_), and ground truth labels corresponding to labeled images (*𝒴*_ℒ_).

In this work, the total loss function follows the definition of

(1) $$\begin{array}{c} L_{\mathrm{total}}\left(G_{\mathrm{IL}},G_{\mathrm{LI}},D_{L},D_{I}\right)=L_{\mathrm{generator}}^{\mathrm{label}}\left(G_{\mathrm{IL}}\right)+\lambda_{1}L_{\mathrm{generator}}^{\mathrm{Image}}\left(G_{\mathrm{LI}}\right)\\ +\lambda_{2}L_{\mathrm{cycle}}^{\mathrm{label}}\left(G_{\mathrm{IL}},G_{\mathrm{LI}}\right)+\lambda_{3}L_{\mathrm{cycle}}^{\mathrm{image}}\left(G_{\mathrm{IL}},G_{\mathrm{LI}}\right)\\ -\lambda_{4}L_{\mathrm{discriminator}}^{\mathrm{label}}\left(G_{\mathrm{IL}},D_{L}\right)-\lambda_{5}L_{\mathrm{discriminator}}^{\mathrm{image}}\left(G_{\mathrm{LI}},D_{I}\right). \end{array}$$

Here, *λ*_*i*_, *i*=1, ⋯, 5 are all constant. Data from [21] suggest that these constants can be set as *λ*_1_=1, *λ*_2_=0.05, *λ*_3_=1, *λ*_4_=1,  and *λ*_5_=1. The object function that boots our network to achieve reasonably accurate sonar targets segmentation from limited labeled data is as follows:

(2) $$\begin{array}{c} \underset{G_{\mathrm{IL}},G_{\mathrm{LI}}}{\mathrm{argmin}}\underset{D_{L},D_{I}}{\mathrm{argmax}}L_{\mathrm{total}}\left(G_{\mathrm{IL}},G_{\mathrm{LI}},D_{L},D_{I}\right). \end{array}$$

**Figure 4:**
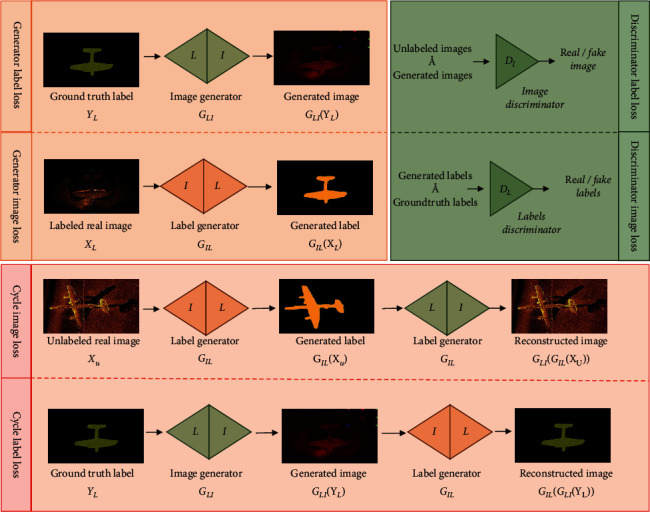
The model contains four networks that are trained simultaneously.

Expression (1) consists of six loss functions of training defined by Mondal [21], which can be classified as three types, generator loss (orange part), discriminator loss/adversarial loss (green part), and cycle-consistency loss (red region), shown in the following expression and Figure 4:

(3) $$\begin{array}{c} \left\{\begin{array}{c} \mathrm{generator loss}\left\{\begin{array}{c} \mathrm{G\_label loss:}\;L_{\mathrm{genenator}}^{\mathrm{label}}\left(G_{\mathrm{IL}}\right),\\ \mathrm{G\_image loss:}\;L_{\mathrm{generator}}^{\mathrm{Image}}\left(G_{\mathrm{LI}}\right), \end{array}\right.\\ \mathrm{discriminnator loss}\left\{\begin{array}{c} D\;\mathrm{\_label}\;\mathrm{loss}\;:L_{\mathrm{discriminator}}^{\mathrm{label}}\left(G_{\mathrm{IL}},D_{L}\right),\\ \mathrm{D\_ image loss:}\;L_{\mathrm{discriminator}}^{\mathrm{Image}}\left(G_{\mathrm{LI}},D_{I}\right), \end{array}\right.\\ \mathrm{cycle-consistency loss}\left\{\begin{array}{c} \mathrm{C \_label loss:}\;L_{\mathrm{cycle}}^{\mathrm{Image}}\left(G_{\mathrm{IL}},G_{\mathrm{LI}}\right),\\ \mathrm{C\_}\;\mathrm{image}\;\mathrm{loss}\;:L_{\mathrm{cycle}}^{\mathrm{label}}\left(G_{\mathrm{IL}},G_{\mathrm{LI}}\right). \end{array}\right) \end{array}\right. \end{array}$$

### 3.3. Spectral Normalization

Previous research has proved that constraining the Lipschitz constant of the discriminator's mapping function *f*(·) can stabilize GAN training [3237]. The reason why applying the spectral normalization to the CycleGAN network can satisfy the L-constraint is given as follows.

For one layer of the fully connected neural network, the definition of the L-constraint is as follows:

(4) $$\begin{array}{c} f\left(Wx_{1}\mathrm{+b}\right)\mathrm{-f}\left(Wx_{2}\mathrm{+b}\right)\leq C\left(\mathrm{W,b}\right)\left\|x_{1}-x_{2}\right\|. \end{array}$$

Here, *f*(·) is the activation function, *W* is the network parameter matrix, *b* is the bias, *C*(*W*, *b*) is a variable about *W* and *b*, and *x*_1_ and *x*_2_ are the input parameters.

When *x*_1_ and *x*_2_ are close enough, equation (4) can be approximated by the first-order term as follows:

(5) $$\begin{array}{c} \left\|\frac{\partial f}{\partial x}W\left(x_{1}-x_{2}\right)\right\|\leq C\left(W,b\right)\left\|x_{1}-x_{2}\right\|. \end{array}$$

Because we use ReLU as the activation function, ‖∂*f*/∂*x*‖=1; equation (5) can be written as follows:

(6) $$\begin{array}{c} \left\|W\left(x_{1}-x_{2}\right)\right\|\leq C\left\|x_{1}-x_{2}\right\|. \end{array}$$

Here, *C* is equal to the spectral norm of the network parameter matrix ‖*W*‖_2_, and the definition is as follows:

(7) $$\begin{array}{c} C={\left\|W\right\|}_{2}=\underset{x\neq 0}{\max}\frac{{\left\|Wx\right\|}_{2}}{{\left\|x\right\|}_{2}}=\sigma \left(W\right). \end{array}$$

Here, *σ*(*W*) is the maximum singular value of *W*.

According to [38], the output and input of the whole network can be written as follows:

(8) $$\begin{array}{c} f\left(x\right)=D_{N}W_{N}\cdots D_{1}W_{1}x{\left\|D_{\text{i}}\right\|}_{2}\leq 1. \end{array}$$

Here, *D*_*i*_(.) is the activation function of each layer, *N* is the number of layers of network, *x* is the input data, and *i* is any single layer in the total *N* layers.

Taking the gradient on both sides of equation (8),

(9) $$\begin{array}{c} {\left\|\nabla_{x}\left(f\left(x\right)\right)\right\|}_{2}={\left\|D_{N}W_{N}\cdots D_{1}W_{1}\right\|}_{2}\\ \leq {\left\|D_{N}\right\|}_{2}{\left\|W_{N}\right\|}_{2}\cdots {\left\|D_{1}\right\|}_{2}{\left\|W_{1}\right\|}_{2}. \end{array}$$

Here, ∇_*x*_ is the gradient operator and ‖*W*‖_2_ is the spectral norm of the network parameter matrix. ‖*D*_i_‖_2_ ≤ 1 because the activation function used in the CycleGAN is ReLU. Therefore, equation (9) can be written as follows:

(10) $$\begin{array}{c} \nabla_{x}{\left(f\left(x\right)\right)}_{2}\leq \prod_{i=1}^{N}\sigma \left(W_{i}\right). \end{array}$$

Finally, both sides of equation (10) are divided by *σ*(*W*_*i*_), namely, spectral normalization, which makes the network satisfy L-constraint:

(11) $$\begin{array}{c} \nabla_{x}{\left(f\left(x\right)\right)}_{2}=D_{N}\left[\frac{W_{N}}{\sigma \left(W_{N}\right)}\right]\cdots D_{1}\left[\frac{W_{1}}{\sigma \left(W_{1}\right)}\right]\leq \prod_{i=1}^{N}\frac{\sigma \left(W_{i}\right)}{\sigma \left(W_{i}\right)}=1. \end{array}$$

It means that spectral normalization of each layer helps *f* of network satisfy the L-constraint.

**Figure 5:**
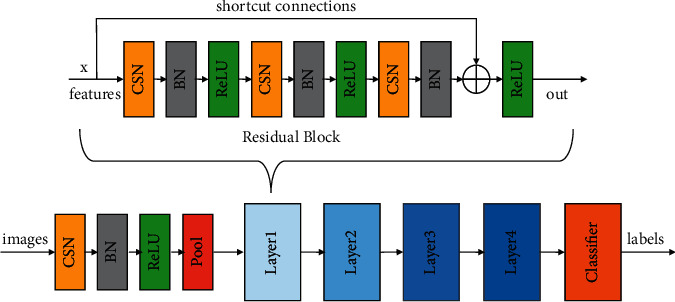
The ResNet of the generators applied with the spectral normalization.

**Figure 6:**
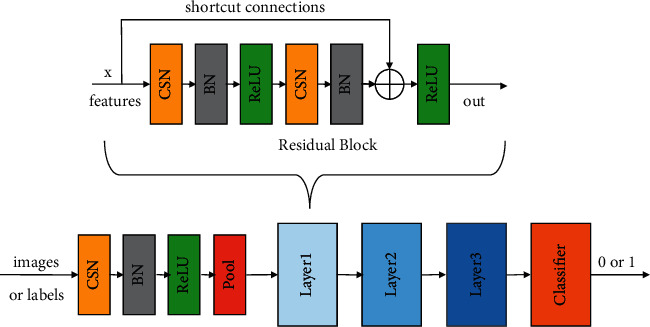
The ResNet of the discriminators applied with the spectral normalization.

The network of the generator and the discriminator which apply spectral normalization is shown in Figures 5 and 6. The architecture is based on ResNet [39], which has four layers. CSN is Conv spectral normalization, which applies spectral normalization to the conv block, BN is batch-normalization, and ReLU is the activation function. Classifier changes the input features into generated labels or images.

## 4. Experiments and Analysis

### 4.1. Sonar Image Datasets

The dataset SCTDI we made is added segmentation labels and dropped too similar images on the basis of the SCTD dataset [8]. It is composed of 300 images, including three categories: aircraft wrecks, shipwrecks, and victims, which are randomly divided into training (270 images) and validation (30 images) subsets.

The dataset SCTDII is acquired from the side-scan sonar Klein series 5000, and the website is “https://www.kleinsonar.com”. It is composed of 800 images of tiny targets, which are randomly divided into training (720 images) and validation (80 images) subsets.

All images have a fixed resolution of 320 × 320 and 9.6 bits per pixel. To reduce the need for memory, the short edges of both datasets fed into the network are shrunken into 200 pixels.

### 4.2. Evaluation Protocol

The mean intersection over union (mIoU) metric [40] is used to evaluate the segmentation results of all the models (supervised model, AdvSemSeg, MT-CutMix, CycleGAN, and ours), which is defined as follows:

(12) $$\begin{array}{c} \text{m}I\text{o}U=\frac{\text{TP}}{\text{TP}+\text{FP}+\text{FN}}, \end{array}$$

where TP, FP, and FN are the true positive, false positive, and false negative pixels, respectively, determined over the whole validation set. The larger the value of mIoU, the better the result of semantic segmentation.

### 4.3. Results

In this section, the training effects of our method on the sonar image datasets SCTDI and SCTDII are firstly described. We also compare the performance of spectral normalization applied to the ResNet with other network structures. Besides, we show the comparison between spectral normalization and the other stabilization methods.

The supervised training results serve as a benchmark using all the labeled images. Three state-of-the-art semisupervised methods and our model are trained on the same training subsets, which are scratched 10%, 20%, 30%, 40%, and 50% of labeled images. All methods are trained without using the pretrained model to have an unbiased comparison.

Tables 1 and 2 compare the semisupervised semantic segmentation accuracy (mIoU/%) of our model with other state-of-the-art methods on SCTDI and SCTDII dataset. The results would seem to suggest that the proposed model outperforms other methods when training with a reduced set of labeled images in all cases. Furthermore, this difference is particularly significant when pixel-level annotations are scarce (i.e., 10% and 20% of the whole training set), where the proposed model achieves 7%–11% of improvement.

**Figure 7:**
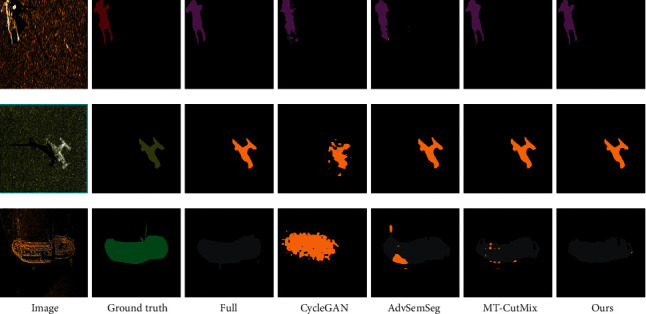
Examples of images, ground truth labels, and generated images from five tested methods: full (the third column), CycleGAN (the fourth column), AdvSemSeg (the fifth column), MT-CutMix (the sixth column), and our model (the seventh column).

**Figure 8:**
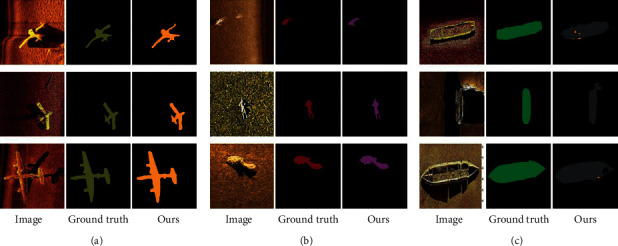
More examples of images, ground truth labels, and generated labels are obtained for three targets: Plane (a), Person (b), and Ship (c).

**Figure 9:**
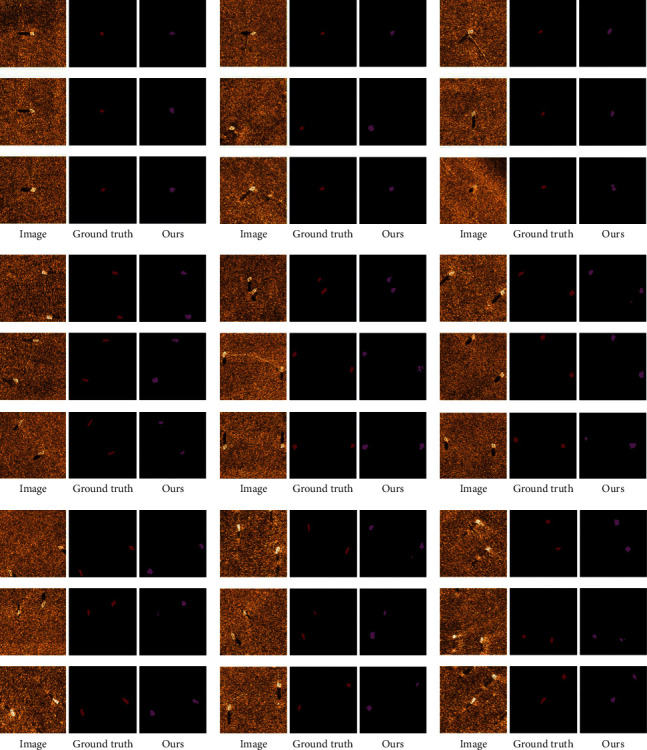
More examples of images, ground truth labels, and generated labels obtained for tiny targets.

The visual comparison of segmentation results is shown in Figure 7. It shows that the proposed method predicts a segmentation closer to the ground truth than other state-of-the-art methods where labeled images are limited. In addition, our model seems to capture better details like legs of persons, wings of planes, and so on. More segmentation results of different shapes and numbers are shown in Figures 8 and 9. Therefore, our approach seems robust when applied to semisupervised segmentation of acoustic images.

Table 3 shows a comparison of the semisupervised semantic segmentation accuracy (mIoU/%) when different network structures are chosen for both the generator and the discriminator. These results would seem to suggest that the spectral normalization does not rely on the different chosen networks and the ResNet has the best performance.

Table 4 shows comparison of the semisupervised semantic segmentation accuracy (mIoU/%) between the spectral normalization and the other stabilization methods. The results prove that the spectral normalization has the best performance and could be a reasonable approach to tackle the issue that limited labeled data are available for segmentation task.

### 4.4. Ablation Study

**Figure 10:**
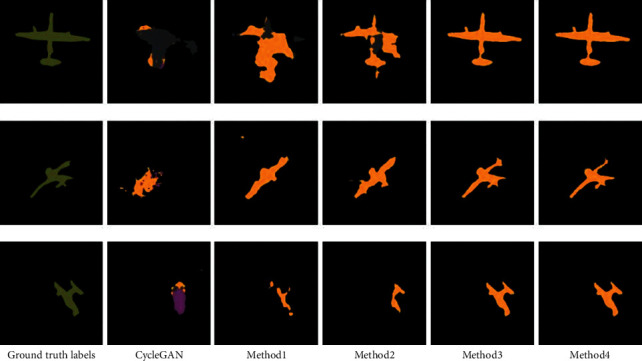
Visual comparisons of different methods on the SCTDI dataset employing 30% of labeled images for training.

To further analyze the effect of the different components of the proposed model, we conduct an ablation study. The results of our ablation study are summarized in Table 5, and the visual comparisons of different methods are in Figure 10. The previous model is CycleGAN. Method 1 refers to applying transfer learning to the CycleGAN. Method 2 refers to applying spectral normalization only to the discriminator. Method 3 refers to applying spectral normalization to both the discriminator and the generator. Method 4 refers to adding a pretrained model to method 3.

The proposed model uses spectral normalization without transfer learning reaches an MIoU value of 0.6437. If we remove the spectral normalization on the generator, this value is reduced to 0.4517. However, removing the spectral normalization on the CycleGAN leads to an even lower accuracy of 0.4138, suggesting that the spectral normalization on segmentation masks has a more substantial impact on the model. Besides, we observe that adding a pretrained model to the proposed model and CycleGAN only helps improve the accuracy of the segmentation results from 0.6437 to 0.6471 and 0.4138 to 0.4229.

## 5. Conclusion

This paper presented an improved semisupervised semantic segmentation method for sonar image based on the CycleGAN network combining spectral normalization. The spectral normalization is applied to both generator and discriminator to solve the problem that the generator tends to generate the same segmentation results when labeled data are limited. According to the experimental results, it has been proved that this strategy can improve the performance of semisupervised segmentation, especially when labeled data are scarce. The segmentation results are robust for underwater objects with different shapes and numbers without transfer learning.

## Figures and Tables

**Table 1:** Semisupervised semantic segmentation performance of different state-of-the-art methods on SCTDI datasets.

Method	The proportion of labels used					
	1/1	½	2/5	3/10	1/5	1/10
Supervised	**0.74**	—	—	—	—	—
AdvSemSeg [41]	—	0.60	0.58	0.40	0.31	0.28
MT-CutMix [35]	—	0.68	**0.66**	0.56	0.37	0.32
CycleGAN [21]	—	0.63	0.61	0.41	0.32	0.27
Ours	—	**0.69**	**0.66**	**0.64**	**0.54**	**0.43**

**Table 2:** Semisupervised semantic segmentation performance of different state-of-the-art methods on SCTDII dataset.

Method	The proportion of labels used					
	1/1	1/2	2/5	3/10	1/5	1/10
Supervised	**0.78**	—	—	—	—	—
AdvSemSeg [41]	—	0.66	0.64	0.59	0.51	0.43
MT-CutMix [35]	—	0.72	0.69	0.63	0.57	0.50
CycleGAN [21]	—	0.67	0.65	0.57	0.53	0.47
Ours	—	**0.75**	**0.70**	**0.68**	**0.65**	**0.61**

**Table 3:** Semisupervised semantic segmentation performance of different networks with spectral normalization on SCTDI datasets.

Network	The proportion of labels used				
	1/2	2/5	3/10	1/5	1/10
LEDNet [42]	0.68	0.64	0.62	0.50	0.42
ENet [43]	0.67	**0.67**	0.63	0.53	0.41
U-Net [44]	0.68	0.65	0.62	0.52	**0.43**
ResNet [39]	**0.69**	0.66	**0.64**	**0.54**	**0.43**

**Table 4:** Semisupervised semantic segmentation performance of different stabilization methods applied to CycleGAN on SCTDII datasets.

Method	The proportion of labels used				
	1/2	2/5	3/10	1/5	1/10
None	0.63	0.61	0.41	0.32	0.27
EB-GAN [31]	0.67	0.62	0.47	0.39	0.32
MRP [33]	0.64	0.61	0.58	0.44	0.37
Gradient penalty [32]	0.63	0.63	0.50	0.42	0.32
SN [38]	**0.69**	**0.66**	**0.64**	**0.54**	**0.43**

**Table 5:** Ablation study on the SCTDI dataset employing 30% labeled data.

Model	mIoU (%)
CycleGAN	0.4138
Method 1	0.4229
Method 2	0.4517
Method 3 (ours)	0.6437
Method 4	0.6471

## Data Availability

The link is “https://github.com/freepoet/SCTD.”

## References

[1] Cervenka P., de Moustier C. (1993). Sidescan sonar image processing techniques. *IEEE Journal of Oceanic Engineering*.

[2] Marx D., Nelson M., Chang E., Gillespie W, Putney A, Warman K An introduction to synthetic aperture sonar.

[3] Putney A., Chang E., Chatham R., Marx D, Nelson M, Warman L. K (2001). Synthetic aperture sonar-the modern method of underwater remote sensing. *IEEE Aerospace Conference Proceedings (Cat*.

[4] Jia Xu, Jiang X., Tang J., Lu L, Zhang J The research of underwater target imaging with high moving sonar based on synthetic aperture method.

[5] Sung M., Lee M., Kim B., Yu S.-C. (2020). Imaging-sonar-based underwater object recognition utilizing object’s yaw angle estimation with deep learning. *IFAC-PapersOnLine*.

[6] Vera J D R, Coiras E., Groen J., Evans B (2009). Automatic target recognition in synthetic aperture sonar images based on geometrical feature extraction. *EURASIP Journal on Applied Signal Processing*.

[7] Petillot Y., Pailhas Y., Sawas J. (2010). *Target Recognition in Synthetic Aperture Sonar and High Resolution Side Scan Sonar Using AUVs*.

[8] Zhang P., Tang J., Zhong H., Ning M, Liu D, Wu K (2021). Self-trained target detection of radar and sonar images using automatic deep learning. *IEEE Transactions on Geoscience and Remote Sensing*.

[9] Karoui I., Fablet R., Boucher J.-M., Augustin J.-M. (2009). Seabed segmentation using optimized statistics of sonar textures. *IEEE Transactions on Geoscience and Remote Sensing*.

[10] Hansen R. E., Callow H. J., Sabo T. O. (2011). Challenges in seafloor imaging and mapping with synthetic aperture sonar. *IEEE Transactions on Geoscience and Remote Sensing*.

[11] Xu H., Zhang L., Er M. J., Yang Q Underwater sonar image segmentation based on deep learning of receptive field block and search attention mechanism.

[12] Rahnemoonfar M., Dobbs D. Semantic segmentation of underwater sonar imagery with deep learning.

[13] Wu M., Wang Q., Rigall E. (2019). ECNet: efficient convolutional networks for side scan sonar image segmentation. *Sensors*.

[14] Yu F., He B., Li K. (2021). Side-scan sonar images segmentation for AUV with recurrent residual convolutional neural network module and self-guidance module. *Applied Ocean Research*.

[15] Goodfellow I. J., Pouget-Abadie J., Mirza M. Generative adversarial nets.

[16] Odena A. (2016). *Semi-supervised Learning with Generative Adversarial Networks*.

[17] Denton E., Gross S., Fergus R. (2016). *Semi-supervised Learning with Context-Conditional Generative Adversarial Networks*.

[18] Han L., Huang Y., Dou H. (2020). Semi-supervised segmentation of lesion from breast ultrasound images with attentional generative adversarial network. *Computer Methods and Programs in Biomedicine*.

[19] Zhu J.-Y., Park T., Isola P., Efros A. A Unpaired image-to-image translation using cycle-consistent adversarial networks.

[20] Jiang J., Hu Y.-C., Tyagi N., Frangi A. F., Schnabel J. A. (2018). Tumor-aware, adversarial domain adaptation from CT to MRI for lung cancer segmentation. *Medical Image Computing and Computer Assisted Intervention – MICCAI 2018*.

[21] Mondal A. K., Agarwal A., Dolz J. (2019). Revisiting CycleGAN for semi-supervised segmentation. *CoRR, abs/1908*.

[22] Everingham M., van Gool L., Williams C. K. I., Winn J., Zisserman A. (2010). The pascal visual object classes (voc) challenge. *International Journal of Computer Vision*.

[23] Cordts M., Omran M., Ramos S. The cityscapes dataset for semantic urban scene understanding.

[24] Bernard O., Lalande A., Zotti C. (2018). Deep learning techniques for automatic MRI cardiac multi-structures segmentation and diagnosis: is the problem solved?. *IEEE Transactions on Medical Imaging*.

[25] Hiasa Y., Otake Y., Takao M. (3/18/2018). *Cross-modality Image Synthesis from Unpaired Data Using CycleGAN: Effects of Gradient Consistency Loss and Training Data Size*.

[26] Huo Y., Xu Z., Bao S. (2017). *Splenomegaly Segmentation using Global Convolutional Kernels and Conditional Generative Adversarial Networks*.

[27] Dong X., Lei Y., Tian S. (2019). Synthetic MRI-aided multi-organ segmentation on male pelvic CT using cycle consistent deep attention network. *Radiotherapy & Oncology*.

[28] Saha S., Bovolo F., Bruzzone L.

[29] Soto Vega P. J., Costa G. A. O. P. d (2021). An unsupervised domain adaptation approach for change detection and its application to deforestation mapping in tropical biomes. *ISPRS Journal of Photogrammetry and Remote Sensing*.

[30] Yang M., Jiao L., Hou B., Liu F., Yang S. (2021). Selective adversarial adaptation-based cross-scene change detection framework in remote sensing images. *IEEE Transactions on Geoscience and Remote Sensing*.

[31] Zhao J., Mathieu M., LeCun Y. (9/11/2016). *Energy-based Generative Adversarial Network*.

[32] Gulrajani I., Ahmed F., Arjovsky M. Improved training of Wasserstein GANs.

[33] Neyshabur B., Bhojanapalli S., Chakrabarti A. (5/23/2017). *Stabilizing GAN Training with Multiple Random Projections*.

[34] Salimans T., Goodfellow I., Zaremba W. (2016). Improved techniques for training gans. *Advances in Neural Information Processing Systems*.

[35] French G., Laine S., Aila T. (2019). *Semi-supervised Semantic Segmentation Needs strong, Varied Perturbations*.

[36] Lai X., Tian Z., Jiang L. Semi-supervised semantic segmentation with directional context-aware consistency.

[37] Grubisic I., Orsic M., Segvic S. A baseline for semi-supervised learning of efficient semantic segmentation models.

[38] Miyato T., Kataoka T., Koyama M. (2018). *Spectral Normalization for Generative Adversarial Networks*.

[39] He K., Zhang X., Ren S. Deep residual learning for image recognition.

[40] Garcia-Garcia A., Orts-Escolano S., Oprea S. (2017). *A Review on Deep Learning Techniques Applied to Semantic Segmentation*.

[41] Hung W.-C., Tsai Y.-H., Liou Y.-T. (2018). *Adversarial Learning for Semi-Supervised Semantic Segmentation*.

[42] Wang Y., Zhou Q., Liu J. (5/7/2019). *LEDNet: A Lightweight Encoder-Decoder Network for Real-Time Semantic Segmentation*.

[43] Paszke A., Chaurasia A., Kim S. (6/7/2016). *ENet: A Deep Neural Network Architecture for Real-Time Semantic Segmentation*.

[44] Ronneberger O., Fischer P., Brox T., Navab N., Hornegger J., Wells W. M. (2015). U-net: convolutional networks for biomedical image segmentation. *Lecture Notes in Computer Science*.

